# Single-Cell Proteomics Analysis of Recurrent Low-Grade Serous Ovarian Carcinoma and Associated Brain Metastases

**DOI:** 10.3389/fonc.2022.903806

**Published:** 2022-05-25

**Authors:** Tanja Pejovic, Pierre-Valérien Abate, Hongli Ma, Jaclyn Thiessen, Christopher L. Corless, Abigail Peterson, Hugues Allard-Chamard, Marilyne Labrie

**Affiliations:** ^1^ Knight Cancer Institute, Oregon Health & Science University, Portland, OR, United States; ^2^ Department of Immunology and Cell Biology, Université de Sherbrooke, Sherbrooke, QC, Canada; ^3^ Department of Obstetrics and Gynecology, Université de Sherbrooke, Sherbrooke, QC, Canada; ^4^ Department of Diagnostic Radiology, Oregon Health & Science University, Portland, OR, United States; ^5^ Service of Rheumatology, Department of Medicine, Université de Sherbrooke, Sherbrooke, QC, Canada

**Keywords:** low-grade serous ovarian cancer, single-cell proteomics, cyclic immunofluorescence, spatial analysis, brain metastases

## Abstract

Between 2% and 6% of epithelial ovarian cancer (EOC) patients develop brain metastases (brain mets), which are incurable and invariably result in death. This poor outcome is associated with a lack of established guidelines for the detection and treatment of brain mets in EOC patients. In this study, we characterize an unusual case of low-grade serous ovarian carcinoma (LGSOC) that metastasized to the brain. Using a spatially oriented single-cell proteomics platform, we compared sequential biopsies of a primary tumor with a peritoneal recurrence and brain mets. We identified several targetable oncogenic pathways and immunosuppressive mechanisms that are amplified in the brain mets and could be involved in the progression of LGSOC to the brain. Furthermore, we were able to identify cell populations that are shared between the primary tumor and the brain mets, suggesting that cells that have a propensity for metastasis to the brain could be identified early during the course of disease. Taken together, our findings further a path for personalized therapeutic decisions in LGSOC.

## Introduction

The incidence of brain metastases (brain mets) in epithelial ovarian cancer (EOC) patients is about 2%–6% and invariably results in death (1). This represents a clinical challenge, since there are currently no established guidelines for the detection and treatment of this severe and irreversible condition (2–7). Previous studies in EOC have shown that brain mets display a unique phenotype and do not respond to systemic therapy in the same way as extracranial tumors (1, 8, 9). This can be explained in part by the inability of some drugs to reach therapeutically relevant concentrations in the brain due to the blood–brain barrier (BBB) and by the brain microenvironment that selectively exerts an evolutionary pressure on the invading cancer cells, thus modifying their phenotype (10–12). The brain tumor microenvironment (TME) is profoundly different from the TME of other organs due to its distinct cell type repertoire, immune cell colonization, a specialized anatomic BBB, and its highly specialized metabolic milieu. Consequently, the brain TME imposes distinct selective pressure on EOC cells and shapes their response to treatment (10–12). Most importantly, studies have clearly shown that brain mets represent a condition for which a specific therapeutic armamentarium is essential.

Low-grade serous ovarian carcinoma (LGSOC) accounts for approximately 3.6% of all ovarian tumor and is characterized by a unique molecular profile and clinical course (13). Abnormalities in Mitogen-activated protein kinase (MAPK) pathway genes are commonly found in LGSOC and include activating mutations in *KRAS* and *BRAF* that act *via* constitutive activation of the MAPK/Extracellular signal-regulated kinase (ERK) pathway (14). The AACR GENIE Cohort of clinical-grade genomic sequencing data generated in eight Clinical Laboratory Improvement Amendments / International Organization for Standardization (CLIA-/ISO)-certified laboratories in the USA revealed the frequency of *KRAS* mutations in LGSOC to be similar to the combined frequencies in cohorts of LGSOC published over the last two decades and represent 27% of the cases (14). The majority of these mutations are in codon 12 of exon 2, and the most frequent variants are KRAS G12V and KRAS G12D. Other mutations with increased frequency are in *BRAF* and *NRAS* genes (14).

Although LGSOC contained to the ovary is associated with a favorable progression-free and overall survival, most LGSOCs are diagnosed at an advanced stage due to lack of specific symptoms and are relatively chemoresistant, leading to a poor outcome (13, 15). Extraperitoneal metastases are very rare, and brain mets are uniquely uncommon. Advanced disease is treated with aggressive surgery that is a cornerstone of treatment, and adjuvant treatment that until recent years included chemotherapy, or anti-estrogen hormonal therapy (13, 15). Most recently, trametinib, an Mitogen-activated protein kinase kinase (MEK) inhibitor, has shown efficacy over chemotherapy and endocrine therapy and has been suggested as the first-line adjuvant treatment for advanced disease (16). To increase response rates, ongoing strategies include combining hormonal treatment with cyclin-dependent kinase 4 and 6 (CDK4/6) inhibitors and exploring the role of Vascular endothelial growth factor (VEGF) inhibitors and immune therapy.

Here, we studied the evolution of an unusual case of LGSOC that metastasized to the brain by performing a detailed spatially oriented single-cell proteomics analysis of brain mets and comparing them with peritoneal disease from the same patient. Importantly, our analyses highlight important pathways that could be involved in the brain metastasis process and reveal several clinically relevant therapeutic vulnerabilities that could inform personalized medicine for LGSOC patients with concomitant brain mets.

## Methods

All tumor samples were obtained in accordance with IRB 3485 protocol at Oregon Health & Science University. All specimens have been evaluated by a board-certified pathologist. A somatic KRAS p.G12D mutation was identified using clinical next-generation sequencing test (GeneTrails^®^ Solid Tumor Panel).

### Histology and Immunofluorescence

LGSOC tissues were fixed in neutral buffered formalin and processed for paraffin embedding. Tissue sections of 5 μm were used for this study. H&E staining was performed by the Histopathology Shared Resource at the Knight Cancer Institute, and whole-slide images were acquired with an Axioscan Z1 (Zeiss) slide scanner.

Cyclic immunofluorescence (Cyc-IF) was performed as previously described by our group (17). Briefly, Cyc-IF allows the detection and spatial single-cell analysis of more than 40 proteins on a single Formalin-Fixed Paraffin-Embedded (FFPE) slide. Multiple sequential rounds of immunofluorescence staining, imaging, and quenching were performed on each sample. The samples were processed as follows: 5-µm FFPE slides were deparaffinized, and antigen retrieval was performed in a Cuisinart pressure cooker (model CPC-600), using pH 6 citrate buffer for 20 min, followed by a quick rinse in distilled water and incubation into pH 9 Tris/Ethylenediamine tetraacetic acid (EDTA) buffer for 15 min. Slides were then blocked in a solution of Phosphate-buffered saline (PBS) with 10% normal goat serum and 1% Bovine Serum Albumin (BSA). The autofluorescence level of each tissue was acquired using an Axioscan fluorescence slide scanner (Zeiss). We then proceeded to sequential staining, imaging, and quenching of each antibody set (a list of primary antibodies used in this study is presented in **Supplementary Table S1**. Briefly, in each cycle, 4 primary antibodies conjugated with Alexa-Fluor 488, 555, 647, or 750 were incubated on the tissue sections for 2 h at room temperature. After washing, each slide was scanned and then quenched in a solution of 3% peroxide and 20 mM NaOH in PBS. After confirming the quenching of the immunofluorescence signal, a new set of antibodies was applied to the slides until all antibodies were sequentially probed.

### Cyclic Immunofluorescence Image Processing and Data Analysis

After all of the images were acquired, registration was performed based on the 4′,6-diamidino-2-phenylindole (DAPI) signal using MATLAB (18). The visualization of the multiplex image, cell segmentation, and feature extraction were performed using QI Tissue Image analysis software. The mean intensities of each marker were extracted from each cell in the appropriate cell compartments. Cells with abnormal features were first filtered based on the nucleus size, and the autofluorescence level acquired in the Alexa-Fluor 555 channel. For each marker, the autofluorescence level acquired at the same wavelength was subtracted on a single-cell basis, and the protein expression values were normalized by a z-score calculation. All heatmaps and clustering analysis were performed using python (github: https://github.com/biodev/cycIF-workflow/tree/v1.0). The spatial analysis of immune cells was performed as follows. Each tumor sample was reconstructed digitally based on the x and y coordinates of the cells. A grid analysis was performed by superposing 150 × 150 pixel squares over the tissue image. In each square, the percentage of each cell type (endothelial, stromal, epithelial, and immune) was calculated. The percentage of each immune cell type was also calculated. To remove areas with no tissue or low cell densities, squares containing 5 cells or less were removed from the analysis. Squares that contained a minimum of 50% epithelial cells were considered as tumor regions. The rest was classified as stroma regions. Furthermore, squares with a minimum of 30% immune cells were considered as immune hot spots.

## Results

### Patient Characteristics

**Figure 1:**
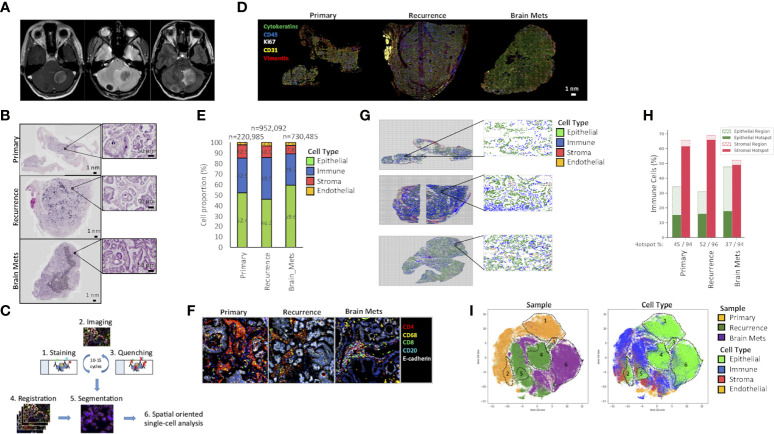
Tumor architecture and composition. **(A)** Brain mets magnetic resonance imaging (MRI). Axial T1 post contrast (left panel) showing two peripherally enhancing masses in the cerebellum. Axial T2* (center panel) demonstrates hypointensity within these masses, indicative of mineralization, and confirmed by hyperdensity on MRI. Axial FLAIR (right panel) with mild hyperintense edema surrounding these masses, particularly the large left cerebellar mass, with mild mass effect on the fourth ventricle. **(B)** H&E staining of the primary ovarian tumor, a pelvic recurrence, and brain mets. **(C)** Cyc-IF framework. For each sample, sequential cycles of staining, imaging, and quenching are performed on a single tissue slide. The images are then aligned through registration, and the segmentation is performed. After extracting the mean intensities of each marker in each cell, a spatially oriented single-cell analysis is performed. **(D)** Immunostaining of epithelial (E-cadherin, cytokeratins), endothelial (CD31), stromal (vimentin), and proliferative (Ki67) markers of the primary ovarian tumor, a pelvic recurrence, and brain mets. **(E)** Tumor composition. The Cyc-IF analysis allows the classification of all cells within epithelial, immune, stromal, and endothelial compartments. The histogram represents the percentage of epithelial, immune, stromal, and endothelial cells in each tumor sample. **(F)** Example of a region enriched in immune cells with the presence of immune “hot spots”. **(G)** Grid analysis. Each tissue analyzed by Cyc-IF was reconstructed using the x and y coordinates of the nucleus. A grid was used to analyze the proportion of each cell type in discrete regions of the tumors. **(H)** For each tumor, the immune cell distribution within the epithelial and stromal compartments was calculated based on the grid analysis. Cells found in aggregates were considered as part of an immune “hot spot”. **(I)** UMAP analysis performed on a subset of 200,000 cells randomly selected across each tumor. Colors represent the samples and the cell type. The full name of each protein can be found in **Supplementary Table S1**.

A 26-year-old woman was diagnosed with a borderline serous tumor of the ovary with micropapillary features after she underwent right salpingo-oophorectomy (RSO). A year later, she underwent additional surgery that included partial removal of the left ovary, and the diagnosis was confirmed. Five years after diagnosis, the patient underwent removal of a 13-cm recurrent pelvic tumor, and pathology confirmed borderline serous tumor with micropapillary features and no invasive implants. A somatic *KRAS* ^G12D^ mutation was identified by next-generation sequencing of tumor from the pelvis. *KRAS* gene mutations are commonly found in LGSOC but not in high-grade serous ovarian carcinoma (HGSOC). A year later, she was diagnosed with multiple brain masses (**Figure 1A**), for which the largest in the left cerebellum was resected. Pathologic analysis revealed an invasive LGSOC. The specimens analyzed in this study included the left ovary (primary) 1 year post diagnosis, recurrent pelvic tumor (recurrence) 5 years post diagnosis, and brain mets 6 years post diagnosis (**Figure 1**). No adjuvant treatment was given prior to emergence of brain mets, per patient’s choice.

### Tumoral Architecture and Composition

The histology of LGSOC, characterized by glandular cells with a low mitotic index [less than 12 mitoses per 10 high-power fields (HPF)] and positive Immunohistochemistry (IHC) staining for WT1 and PAX8, was confirmed by a board-certified pathologist. As LGSOC can present with various architectural patterns, including compact cell nests, cribriform, and micropapillary and glandular structures (13), we assessed changes in the tumor’s architecture patterns during progression. An H&E coloration of the samples (**Figure 1B**) revealed the pelvic recurrence, and brain mets harbored a more invasive phenotype compared to the primary tumor. The primary tumor presented as a serous borderline tumor of low malignant potential with micropapillary architecture but no invasion of surrounding tissue. The pelvic recurrence was a serous borderline tumor of low malignant potential with transformation into LGSOC. Immune microinvasion was characterized by the presence of eosinophilic cell clusters with a fibrous core on large papillae (foci of <5 mm). Additional complex proliferation of variably sized papillae with mild nuclear atypia and occasional mitoses was also observed. Characteristics of invasive low-grade ovarian carcinoma were observed, including inverted micropapillae, micropapillae with no stroma, and compact cell nests that consisted of small clusters of well-differentiated cells in fibrous stroma that was surrounded by clear spaces. Stromal and capsular invasion was also present. The brain mets showed similar histopathological features to the recurrent tumor. These tumor cells retained low-grade nuclear features with frequently ciliated apical borders, and the number of mitoses remained low at 4/10 HPF. At the interface with cerebellar tissue, small clusters of cells showed invasion into the brain tissue.

To characterize the evolution in the tumor composition during progression from a localized tumor to brain metastasis, single-cell proteomics analysis was performed using multiplex Cyc-IF, as previously described (17). By performing sequential cycles of staining, imaging, and quenching, Cyc-IF allowed the visualization and spatially oriented analysis of 42 proteins at the single-cell level in a single tissue slide of each sample (19) (**Figure 1C**). First, markers that are specific to epithelial, immune, endothelial, and stromal cells were used to determine the cell composition of each tumor sample (**Figure 1D**; **Supplementary Table S1**). As shown in **Figure 1E**, immune and stromal cell populations were slightly decreased in the brain mets (immune: 29.7%, stromal: 7.8%) compared to the primary tumor (immune: 32.9%, stromal: 12.5%) and the recurrence (immune: 39.5%, stromal: 10.9%). In all samples, immune cells were located mostly in the stroma rather than in contact with tumor cells. Some of them were also found clustered together, forming immune hot spots in the tumor stroma (**Figure 1F**). These immune hot spots were composed of CD4+ lymphocytes and CD68+ macrophages. In the brain mets however, these clusters also included CD8+ T cells and CD20+ B cells, consistent with changes in the tumor immune landscape. In order to investigate the spatial organization of the immune cells and the presence of immune hot spots, a grid analysis was performed (**Figure 1G**). Counting the number of cells in tumor and stromal regions of the grid indicated that in the tumor masses analyzed, ~95% of the immune cells found in the stroma were aggregated together in hot spots. While the primary and recurrent tumor had similar proportions of immune cells infiltrating the tumor cell areas (30%–35%), a larger proportion of the brain metastasis immune cells were found in association with the tumor cells (48%). Furthermore, immune cells in the brain mets tended to be more dispersed throughout the specimen, rather than forming immune cell aggregates. Although the role of immune hot spots in the antitumor response in ovarian cancer has yet to be explored, non-uniform distribution of immune cells in the tumor has previously been associated with a differential selective pressure in tumor regions, which can lead to increased tumor heterogeneity (20).

To assess tumoral heterogeneity, a Uniform Manifold Approximation and Projection (UMAP) clustering analysis was performed. As shown in **Figure 1H**, most of the cells clustered by sample, indicating distinct features that are tumor lesion dependent. The brain metastasis tumor cells were concentrated into one main cluster (region #6), while the primary and pelvic recurrence tumor cells clustered in two main populations each (Primary: #1–#2; Recurrence: #4–#5) and one smaller shared region (region #3). Immune endothelial and stromal cells clustered in many populations, which could be attributed to the different cell types targeted by our antibody panel and cell localization (stroma, stroma–epithelial transition, and epithelial compartments). Of note, brain mets and the recurrent tumor cell populations clustered closer together than with the primary tumor, suggesting similarities in the composition of the metastatic TME.

### Tumor Cell Heterogeneity Is Decreased in Brain Metastases

**Figure 2:**
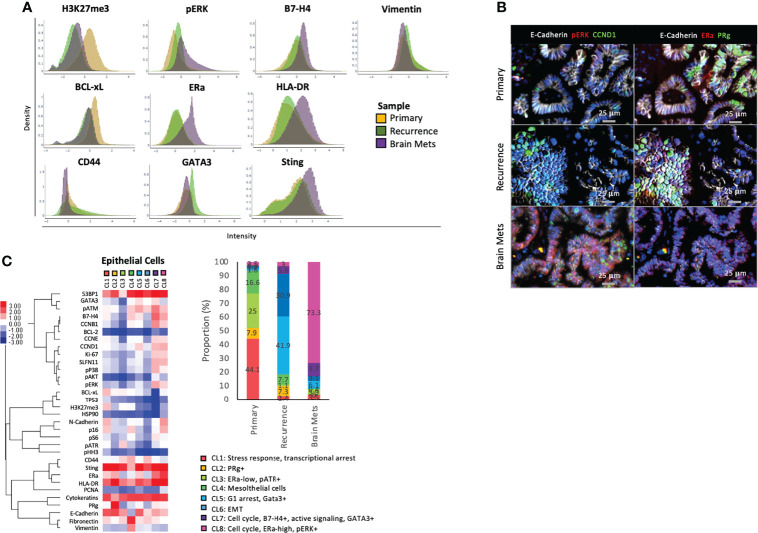
Epithelial cell phenotype. **(A)** Density plot showing the distribution of expression of specific markers across epithelial cells from each sample. **(B)** Representative immunostaining of markers that are differentially expressed across samples. **(C)** A K-Mean clustering was performed on epithelial cells. The heat maps represent the median expression of each marker within each cluster (CL), and the phenotype of each cluster is annotated. The histogram represents the frequency of each cluster within the samples, and the cluster phenotypes are described below the histogram. The full name of each protein can be found in **Supplementary Table S1**.

To characterize the difference between cell populations across samples (cancer cells, immune, and stromal), we compared protein expression profiles using Cyc-IF. For cancer cells (**Figures 2A, B**), we found that the primary disease expressed higher levels of the transcriptional repressor H3K27me3 and antiapoptotic protein BCL-xL, which could be attributed to a stress response (21). Cancer cells in the recurrence displayed a phenotype of epithelial–mesenchymal transition (EMT), as evidenced by increased expression of mesenchymal proteins CD44 and vimentin (22, 23). GATA3, which has been associated with tumor progression in ovarian cancer, was also increased (24). In the brain mets, we observed an increased expression of Sting, a potent inducer of interferon, and its downstream effector Human Leukocyte Antigen – DR isotype (HLA-DR). Importantly, both are known to be involved in the antitumor immune response (25, 26). The brain mets also harbor increased B7-H4 expression, a mediator of T-cell suppression that is thought to behave as an immune checkpoint inducer. This member of the B7 superfamily has been previously associated with tumor immune escape (27) and T-cell suppression (28). Finally, the brain mets had higher expression of Estrogen receptor alpha (ERα and phosphorylation of the MAPK signaling intermediate ERK1/2. Interestingly, GATA3 that is a target of Estrogen receptor alpha (ERα) remained low, indicating that ERα might target genes through a non-canonical pathway (29). Importantly, non-canonical estrogen signaling has been previously associated with resistance to endocrine therapy, which is an important management option for LGSOC (30). Taken together, the overall protein expression pattern in epithelial cells suggested an increased invasive phenotype of the recurrent tumor and increased oncogenic signaling and altered immunogenic phenotype of the brain mets compared to the primary tumor.

To delineate tumor epithelial cell heterogeneity, we performed a K-mean clustering using the single-cell data from the epithelial compartment of each tumor (**Figure 2C**). A total of 8 clusters representing different cell phenotypes were characterized and quantified across the tumor samples. CL1, CL3, and CL4 were almost exclusively found in the primary tumor and represented populations with active stress responses and transcriptional arrest (CL1: high H3K27me3, BCL-xL, p16, pATM, low Ki67), low ERα expression, and elevated phosphorylation of the DNA damage response protein ATR (31) (CL3), as well as a population of mesothelial cells [CL4: Cytokeratins (CKs) and vimentin-high, E-cadherin-low]. CL2, which was absent from the brain mets, was only detected in the primary (7.9% of total epithelial cells) and recurrence (7.3% of total epithelial cells) and was characterized by increased expression of the progesterone receptor (PRg). CL5 and CL6 were found mostly in the recurrence and represented the population of cells with G1 arrest and high GATA3 expression (CL5) and cells with an EMT phenotype (CL6: low CKs and E-cadherin and high vimentin and fibronectin). CL7, which represented cells with an active cell cycle (CCND1, CCNB1, CCNE, and/or Ki67 expression), oncogenic signaling pathways (increased pERK, pS6, and pAKT), and high GATA3 and B4-H7 were found in each sample, but with a predominance in the brain mets (primary 0.6%, recurrence 5.6%, and brain mets 9.7% of total epithelial cells). Finally, CL8 was mostly found in the brain mets and was its main cell population (73.3% compared to 2.5% in the primary and 7.33% in the recurrence). This cluster was enriched in cells that expressed cell cycle proteins (CCND1, CCNB1, Ki67, CCNE) and a high level of ERα and phosphorylation of ERK1/2. The expression of GATA3 remained low, confirming our previous observation of a low canonical signaling pathway activity downstream of ERα. Taken together, this clustering analysis demonstrated that although most cell populations were shared across all samples, the frequency of these populations was markedly altered in brain mets. These mets displayed a more homogeneous pattern, with the predominance of cluster CL8, that could be a result of selective pressure from the brain microenvironment.

### Increased Heterogeneity and Oncogenic Signaling Pathway Activity in the Stromal Compartment of Brain Metastases

**Figure 3:**
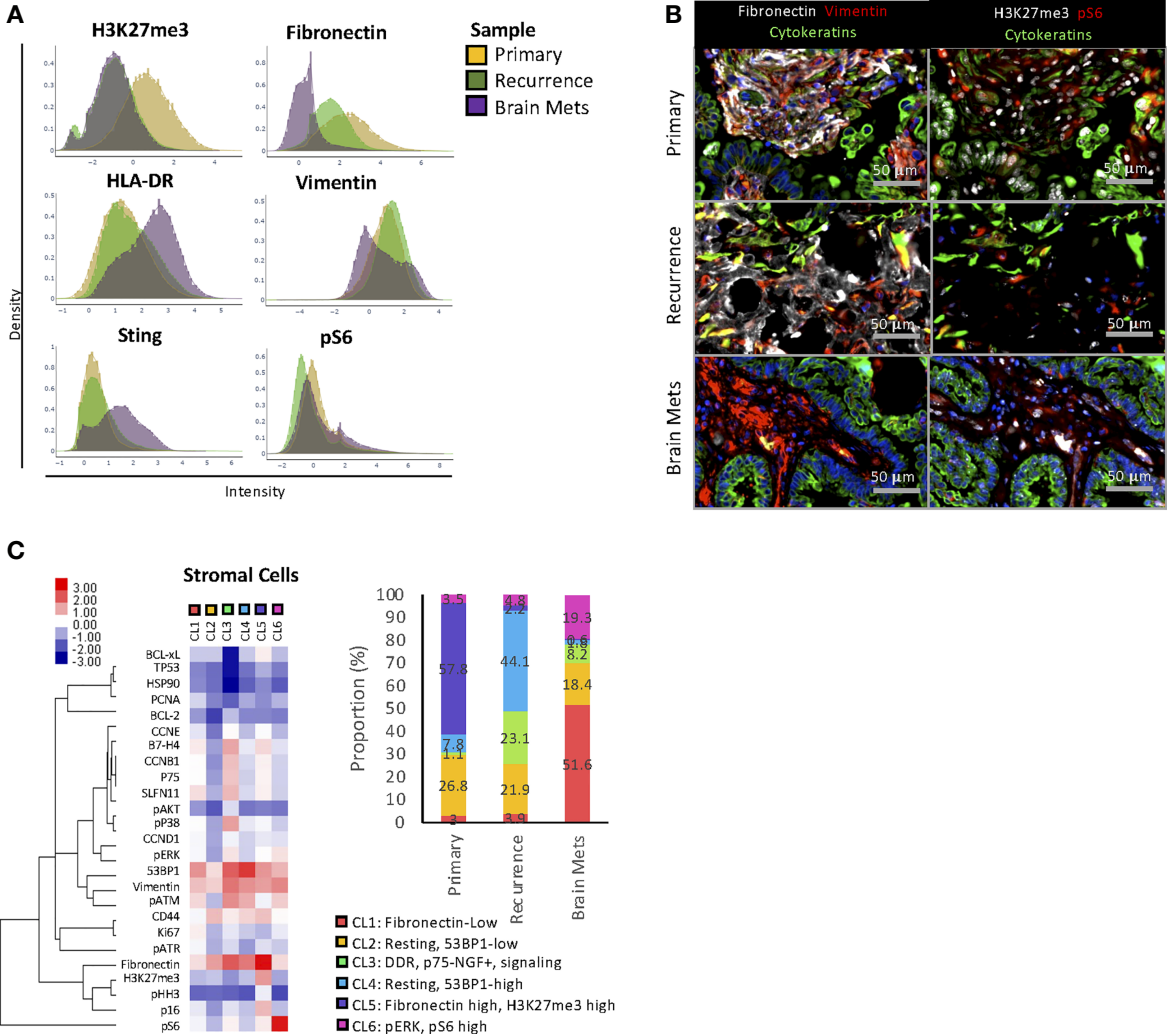
Stromal cell phenotype. **(A)** Density plot showing the distribution of expression of specific markers across stromal cells from each sample. **(B)** Representative immunostaining of markers that are differentially expressed across samples. **(C)** A K-Mean clustering was performed on stromal cells. The heat maps represent the median expression of each marker within each cluster (CL), and the phenotype of each cluster is annotated. The histogram represents the frequency of each cluster within the samples, and the cluster phenotypes are described below the histogram. The full name of each protein can be found in **Supplementary Table S1**.

The protein expression analysis demonstrated that stromal cells displayed different protein expression profiles across tumors. In the brain mets, a decreased expression of fibronectin and vimentin was observed in the stromal compartment compared to the peritoneal tumors, indicating a different composition of the TME (**Figures 3A, B**). Furthermore, this sample expressed a higher level of immunostimulatory proteins HLA-DR and Sting, as well as an increased phosphorylation of ribosomal protein S6, suggesting enhanced Mammalian target of rapamycin complex (mTORC) activity (32). Conversely, stromal cells from the primary tumor displayed a higher level of the transcriptional repressor H3K27me3. In fibroblasts, H3K27me3 has been reported as a negative regulator of fibroblastic activation and transformation into cancer-associated fibroblasts (CAFs) (33, 34).

To identify changes in stromal cell heterogeneity, K-mean clustering analysis (**Figure 3C**) was performed. Among the 6 clusters identified, CL2 and CL5 were the main clusters found in the primary tumor. CL2 represented resting and 53BP1-low cells, while CL5 was enriched in H3K27me3 and fibronectin-high cells. The recurrence was mostly composed of CL2, CL3, and CL4. CL3 was enriched in cells with active DNA damage response (DDR) and signaling pathways (p-ERK1/2, p-AKT moderate, p-S6) and high expression of B7-H4 and p75-nerve growth factor receptor (p75-NGFR). Of note, p75-NGFR has been previously associated with local recurrence and tumor metastasis (35). CL4 was enriched in resting 53BP1-high cells. The brain mets had a markedly different stromal composition compared to the peritoneal disease, with an enrichment in cells from CL1 (fibronectin-low), CL2, CL3, and CL6 (pERK and pS6-high). Aside from CL2 that was shared with similar proportions in each tumor and CL3 that was present in both the recurrent pelvic tumor and the brain mets, the clustering analysis demonstrated that the stromal composition was tumor location dependent. Furthermore, as expected, the brain metastasis stromal composition was vastly different from that of peritoneal disease, indicating a unique TME.

### Altered Immune Landscape in Brain Metastases

**Figure 4:**
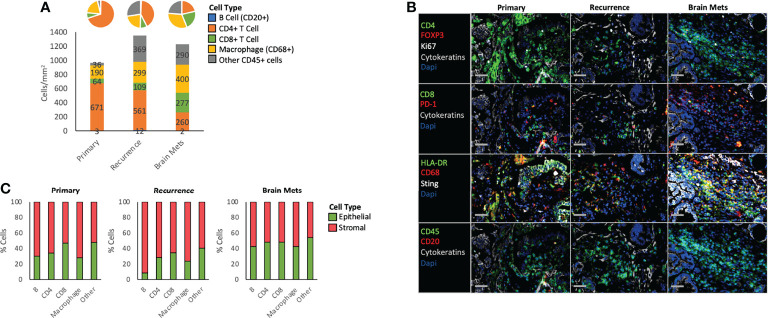
Immune phenotype. **(A)** Histogram and pie chart showing the density and the proportion of different immune cell populations across all samples. **(B)** Representative immunostaining of specific markers within each sample. **(C)** Proportion of each immune cell subtype in the epithelial and the stromal compartments. The percentages were calculated based on the grid analysis. The full name of each protein can be found in **Supplementary Table S1**.

To determine the immune composition of each sample, the immune cells were classified and quantified using Cyc-IF and classic lineage-defining markers (**Figures 4A, B**). We first determined the density of each immune cell population by measuring the number of cells per mm^2^ of tissue. Notably, while B cells were underrepresented, the immune cell density increased in the recurrence and the brain mets, with a decrease in the density of CD4+ T cells and increase in CD68+ macrophages. Additionally, the CD8+ T cell population increased slightly in the recurrence (109 cells/mm^2^) and more drastically in the brain mets, reaching a density of 277 cells/mm^2^, which is considered a “hot” tumor (36). Furthermore, a large proportion of other CD45+ cells were found in the recurrence (369 cells/mm^2^) and the brain mets (290 cells/mm^2^). In terms of immune spatial organization, grid analysis (**Figure 4C**) showed little difference between the stromal and epithelial distribution of immune cells in the primary and recurrence tumor, except for B cells that tended to be more stroma-localized in the recurrence compared to the primary tumor. In the brain mets, an increased proportion of B cells, CD4 T cells, and macrophages were observed in the epithelial compartment when compared to the primary tumor. The CD8 T cells, which are the main effector of the antitumor immune response, remained unchanged, indicating the possible activation of immunosuppressive mechanisms in the TME. By further characterizing these immune cell populations (**Table 1**), we found that Programmed cell death protein 1 (PD-1) immune checkpoint protein expression was increased in the recurrence and the brain mets on both B cells (primary: 0%, recurrence: 1.9%, brain mets: 4.9%), indicating an immunosuppressive phenotype (37), and CD8+ T cells (primary: 0.8%, recurrence: 1.9%, brain mets: 3.6%), in line with an exhausted T-cell phenotype (38). Regulatory T cells (Tregs) (CD4+ FOXP3+) were also more abundant in the recurrence and the brain mets (primary: 0.2%, recurrence: 0.4%, brain mets: 0.5%), which could also contribute to immune evasion. CD44, which is a marker of T-cell activation and memory T cells, was massively decreased in the pelvic recurrence and the brain mets compared to the primary tumor in both CD4+ (primary: 84.3%, recurrence: 40.7%, brain mets: 32.5%) and CD8+ (primary: 63.4%, recurrence: 44.7%, brain mets: 27.5%) T cells. On the other hand, Ki67 staining indicated a higher proliferation rate of B cells (primary: 3.5%, recurrence: 2%, brain mets: 6.7%) and CD8+ T cells in the brain mets (primary: 2.1%, recurrence: 3.1%, brain mets: 6.2%). The interferon pathways encompassing Sting and HLA-DR immunostimulatory molecules were also increased in brain mets. Taken together, this analysis demonstrates that the immune landscape has been profoundly altered during tumor progression. Although a higher immune infiltration has been observed in the brain mets, several immunosuppressive molecules were increased concomitantly, which could contribute to tumor immune escape.

**Table 1:** Immune cell phenotypes.

Phenotype	Cell Type	Primary (%)	Recurrence (%)	Brain Mets (%)
PD-1+	B cell	0.0	1.9	4.9
	CD4 T cells	0.3	0.6	0.3
	CD8 T cells	0.8	1.9	3.6
	Macrophages	0.1	0.2	0.3
	Other CD45+ cells	0.7	0.2	0.2
Tregs (FOXP3+)	CD4 T cells	0.2	0.4	0.5
Ki67+	B cell	3.5	2.0	6.7
	CD4 T cells	2.3	2.9	2.3
	CD8 T cells	2.1	3.1	6.2
	Macrophages	1.6	2.5	2.2
	Other CD45+ cells	3.1	4.2	2.0
CD44-High	CD4 T cells	84.3	40.7	32.5
	CD8 T cells	63.4	44.7	27.5
HLA-DR-High	B cell	6.0	21.0	36.8
	CD4 T cells	9.7	26.4	38.5
	CD8 T cells	6.6	8.1	23.8
	Macrophages	23.8	54.6	58.4
	Other CD45+ cells	4.2	9.8	23.9
Sting-High	B cell	31.0	34.3	38.0
	CD4 T cells	11.5	14.6	54.3
	CD8 T cells	17.8	28.9	52.0
	Macrophages	7.6	17.3	26.5
	Other CD45+ cells	22.9	30.5	61.4

The full name of each protein can be found in **Supplementary Table S1**.

## Discussion

The main objective of this study was to characterize the histopathologic progression of an unusual case of LGSOC from local disease to pelvic recurrence and subsequent metastasis to the brain, with the goal of uncovering therapeutic vulnerabilities that could be exploited in the treatment of patients with EOC and brain mets. This represents the first spatially oriented single-cell proteomics analysis of the progression of an LGSOC tumor using sequential biopsies acquired from the same patient. Our spatially oriented single-cell proteomics analysis showed a major remodeling of the epithelial, stromal, and immune compartments in the brain mets compared to the peritoneal tumors. This remodeling is accompanied by activation of oncogenic prosurvival signaling pathways and increased estrogen receptor expression. Additionally, we observed a profound alteration of the immune landscape and dysregulation of the balance between protumoral and antitumoral immune pathways.

The brain microenvironment can exert selective pressure on cancer cells and shape their response to therapy (10–12), but there is a gap in knowledge about the functional programs used by ovarian cancer cells to thrive in that environment. Furthermore, there is little understanding about the EOC brain metastatic niche itself and how it supports metastasis survival and growth. Since proteins are the functional unit of the cell, single-cell proteomics analysis represents a unique (both high-grade and low-grade serous) opportunity to characterize tumor composition and architecture and define the activity of targetable oncogenic pathways. Because of the high resolution that can be achieved with Cyc-IF, we were able to demonstrate a major remodeling of the epithelial, stromal, and immune compartments. Interestingly, the phenotype of the brain mets was less heterogeneous than in the recurrent peritoneal disease. It could be a result of the selective pressure from the brain microenvironment on the invading cells and highlighting the phenotypic features required for the survival of ovarian cancer cells in that environment. We saw a complete absence of cells that express PR, but concomitantly, all cells expressed a high level of ERα in the brain mets. It is unclear if this change in hormonal receptor profile is due to a change in tumor cell phenotypes or if it is an indication that only certain clones from the peritoneal disease were able to disseminate to the brain. In all cases, cancer cells from the brain mets adopted a prosurvival phenotype, showed by increased MAPK and Phosphoinositide 3-kinase (PI3K) pathway activity. While the frequency of each cancer cell population varied from one tumor to another, some cell phenotypes were shared across the three tumors, suggesting that cells with a propensity for brain invasion and colonization are already detectable in the primary tumor at the time of diagnosis. This supports the hypothesis that brain mets can be established early during the course of disease but may take a long period of time to adapt to the brain microenvironment and progress to cause symptoms. This hypothesis is also supported by the clinical impression that brain mets have become slightly more frequent with the improvement in the systemic treatment of EOC (2–7) and the increase in overall survival. Thus, confirming this hypothesis in a larger cohort of patients with EOC brain mets will be critical to identify and implement effective prevention and therapeutic strategies against EOC brain mets.

In regard to the microenvironment in brain mets, we observed reduced stromal content with major changes in immune cell populations. In contrast with the cancer cells, the stromal cell populations remained as heterogeneous in the brain mets as in the peritoneal tumors but harbored a more “active” phenotype, with increased MAPK and mTORC signaling pathway activity. This was accompanied by reduced expression of vimentin and fibronectin, which are both known to affect cell motility and metastasis in many cancer models (39, 40). As the peritoneal tumors displayed high levels of fibronectin and vimentin in the stromal space, it is possible that the stroma composition facilitated the dissemination of cancer cells to the brain, but once established, high levels of vimentin and fibronectin were not essential for survival of the brain mets. Importantly, the immune landscape was also greatly altered in the brain mets compared to the peritoneal tumors. Indeed, we observed many signs of an antitumor immune contexture such as increased Sting pathway activity and increased CD8+ T cells. However, immunosuppressive cells populations also increased concurrently. Indeed, macrophage density doubled compared to the primary tumor, a larger proportion of B cells and C8+ T cells expressed the immune checkpoint protein PD-1, and the Treg population increased. This change in balance in protumor and antitumor immune response is consistent with ongoing immune activation within the brain mets triggering immunomodulation that can eventually lead to exhaustion/dysfunction of the T cells. Importantly, several studies have tested the efficacy of immune checkpoint blockade (ICB) for the treatment of patients with brain mets. In melanoma and non-small cell lung cancer, for example, ICB is associated with clinical benefit against brain mets (41). Thus, this could also be true for EOC patients with brain mets and could represent a therapeutic opportunity.

In conclusion, our results suggest that brain mets of LGSOC have a unique phenotype compared to that of the peritoneal tumors, which support the premise that standard of care might not be appropriate for the treatment of brain mets. The unique milieu in which brain mets grow can exert selective pressure on the cancer cells, leading to the induction of prosurvival oncogenic pathways and immunosuppressive mechanisms. Thus, it will be important in future studies to obtain a better understanding of the targetable pathways that the cancer cells use for their invasion and survival in the brain microenvironment. Furthermore, as the BBB restricts the ability of certain drugs to reach therapeutically significant concentrations in the brain, it is crucial that we develop novel personalized therapeutic approaches for the treatment of patients with ovarian cancer while considering the ability of the drugs to reach therapeutic concentrations in the brain microenvironment. Although in this case ovarian cancer displayed molecular and clinical features of LGSOC, the wealth of information obtained from single-cell spatial proteomics analyses of primary tumor and brain mets proves that the analyses are likely to be of importance when applied to high-grade serous EOC and is likely to contribute to discerning the mechanisms of brain mets in all EOCs.

## Data Availability Statement

The raw data supporting the conclusions of this article will be made available by the authors without undue reservation.

## Ethics Statement

The studies involving human participants were reviewed and approved by IRB 3485 protocol at Oregon Health & Science University. The patients/participants provided their written informed consent to participate in this study.

## Author Contributions

ML and TP have conceptualized the study and wrote the article. P-VA and HM contributed to the Cyc-IF assay. CC and JT contributed to the pathology and MR imaging, respectively. AP contributed to sample identification, literature search, and article writing. HA-C contributed to the immune monitoring data analysis. All authors contributed to the article and approved the submitted version.

## Funding

This project was supported by the Adelson Medical Research Foundation, the Sherie Hildreth Ovarian Cancer Foundation. P-VA is supported by Grant 878491 from the Cancer Research Society and by Ovarian Cancer Canada/OvCAN through funding provided by Health Canada.

## Conflict of Interest

The authors declare that the research was conducted in the absence of any commercial or financial relationships that could be construed as a potential conflict of interest.

## Publisher’s Note

All claims expressed in this article are solely those of the authors and do not necessarily represent those of their affiliated organizations, or those of the publisher, the editors and the reviewers. Any product that may be evaluated in this article, or claim that may be made by its manufacturer, is not guaranteed or endorsed by the publisher.

## References

[1] Borella F Bertero L Morrone A Gambella A Bovetti M Cosma S. Brain Metastases From Ovarian Cancer: Current Evidence in Diagnosis, Treatment, and Prognosis. Cancers (Basel) (2020) 1:22–12. doi: 10.3390/cancers12082156 PMC7464214 32759682

[2] Cohen ZR Suki D Weinberg JS Marmor E Lang FF Gershenson DM. Brain Metastases in Patients With Ovarian Carcinoma: Prognostic Factors and Outcome. J Neurooncol (2004) 66:313–25. doi: 10.1023/b:neon.0000014516.04943.38 15015663

[3] Cormio G Loizzi V Falagario M Calace A Colamaria A De Tommasi A. Central Nervous System Metastases From Epithelial Ovarian Cancer: Prognostic Factors and Outcomes. Int J Gynecol Cancer (2011) 21:816–21. doi: 10.1097/IGC.0b013e318216cad0 21613959

[4] D'Andrea G Roperto R Dinia L Caroli E Salvati M Ferrante L. Solitary Cerebral Metastases From Ovarian Epithelial Carcinoma: 11 Cases. Neurosurg Rev (2005) 28:120–3. doi: 10.1007/s10143-004-0363-4 15558348

[5] Ratner E Bala M Louie-Gao M Aydin E Hazard S Brastianos PK. Increased Risk of Brain Metastases in Ovarian Cancer Patients With BRCA Mutations. Gynecol Oncol (2019) 153:568–73. doi: 10.1016/j.ygyno.2019.03.004 30876674

[6] Stasenko M Cybulska P Feit N Makker V Konner J O'Cearbhaill RE. Brain Metastasis in Epithelial Ovarian Cancer by BRCA1/2 Mutation Status. Gynecol Oncol (2019) 154:144–9. doi: 10.1016/j.ygyno.2019.05.004 PMC6589378 31113680

[7] Xi S Li Z Guo Q Lin W Liang X Ma L. Prognostic Factors Among Brain Metastases in Newly Diagnosed Ovary Cancer: A Large Real-World Study. J Cancer (2020) 11:4625–40. doi: 10.7150/jca.44494 PMC7255353 32489480

[8] Pakneshan S Safarpour D Tavassoli F Jabbari B. Brain Metastasis From Ovarian Cancer: A Systematic Review. J Neurooncol (2014) 119:1–6. doi: 10.1007/s11060-014-1447-9 24789253

[9] McMeekin DS Kamelle SA Vasilev SA Tillmanns TD Gould NS Scribner DR. Ovarian Cancer Metastatic to the Brain: What is the Optimal Management? J Surg Oncol (2001) 78:194–200. doi: 10.1002/jso.1149 11745806

[10] Fortin D. The Blood-Brain Barrier: Its Influence in the Treatment of Brain Tumors Metastases. Curr Cancer Drug Targets (2012) 12:247–59. doi: 10.2174/156800912799277511 22229251

[11] Suh JH Kotecha R Chao ST Ahluwalia MS Sahgal A Chang EL. Current Approaches to the Management of Brain Metastases. Nat Rev Clin Oncol (2020) 17:279–99. doi: 10.1038/s41571-019-0320-3 32080373

[12] Fares J Cordero A Kanojia D Lesniak MS. The Network of Cytokines in Brain Metastases. Cancers (Basel) (2021) 13(1):17–13. doi: 10.3390/cancers13010142 PMC7795138 33466236

[13] Gadducci A Cosio S. Therapeutic Approach to Low-Grade Serous Ovarian Carcinoma: State of Art and Perspectives of Clinical Research. Cancers (Basel) (2020) 12(1):13–12. doi: 10.3390/cancers12051336 PMC7281204 32456205

[14] Moujaber T Balleine RL Gao B Madsen I Harnett PR DeFazio A. New Therapeutic Opportunities for Women With Low-Grade Serous Ovarian Cancer. Endocr Relat Cancer (2021) 29:R1–R16. doi: 10.1530/ERC-21-0191 34636747

[15] Angarita AM Cholakian D Fader AN. Low-Grade Serous Carcinoma: Molecular Features and Contemporary Treatment Strategies. Expert Rev Anticancer Ther (2015) 15:893–9. doi: 10.1586/14737140.2015.1052411 26040191

[16] Gershenson DM Okamoto A Ray-Coquard I. Management of Rare Ovarian Cancer Histologies. J Clin Oncol (2019) 37:2406–15. doi: 10.1200/JCO.18.02419 31403866

[17] Labrie M Li A Creason A Betts C Keck J Johnson B. Multiomics Analysis of Serial PARP Inhibitor Treated Metastatic TNBC Inform on Rational Combination Therapies. NPJ Precis Oncol (2021) 5:92. doi: 10.1038/s41698-021-00232-w 34667258 PMC8526613

[18] Eng J Thibault G Luoh SW Gray JW Chang YH Chin K. Cyclic Multiplexed-Immunofluorescence (cmIF), a Highly Multiplexed Method for Single-Cell Analysis. Methods Mol Biol (2020) 2055:521–62. doi: 10.1007/978-1-4939-9773-2_24 31502168

[19] Lin JR Fallahi-Sichani M Sorger PK. Highly Multiplexed Imaging of Single Cells Using a High-Throughput Cyclic Immunofluorescence Method. Nat Commun (2015) 6:8390. doi: 10.1038/ncomms9390 26399630 PMC4587398

[20] Gonzalez H Hagerling C Werb Z. Roles of the Immune System in Cancer: From Tumor Initiation to Metastatic Progression. Genes Dev (2018) 32:1267–84. doi: 10.1101/gad.314617.118 PMC6169832 30275043

[21] Braun F de Carne Trecesson S Bertin-Ciftci J Juin P. Protect and Serve: Bcl-2 Proteins as Guardians and Rulers of Cancer Cell Survival. Cell Cycle (2013) 12:2937–47. doi: 10.4161/cc.25972 PMC3875667 23974114

[22] Xu H Tian Y Yuan X Wu H Liu Q Pestell RG. The Role of CD44 in Epithelial-Mesenchymal Transition and Cancer Development. Onco Targets Ther (2015) 8:3783–92. doi: 10.2147/OTT.S95470 PMC4689260 26719706

[23] Liu CY Lin HH Tang MJ Wang YK. Vimentin Contributes to Epithelial-Mesenchymal Transition Cancer Cell Mechanics by Mediating Cytoskeletal Organization and Focal Adhesion Maturation. Oncotarget (2015) 6:15966–83. doi: 10.18632/oncotarget.3862 PMC4599250 25965826

[24] El-Arabey AA Denizli M Kanlikilicer P Bayraktar R Ivan C Rashed M. GATA3 as a Master Regulator for Interactions of Tumor-Associated Macrophages With High-Grade Serous Ovarian Carcinoma. Cell Signal (2020) 68:109539. doi: 10.1016/j.cellsig.2020.109539 31935430

[25] Axelrod ML Cook RS Johnson DB Balko JM. Biological Consequences of MHC-II Expression by Tumor Cells in Cancer. Clin Cancer Res (2019) 25:2392–402. doi: 10.1158/1078-0432.CCR-18-3200 PMC6467754 30463850

[26] Jiang M Chen P Wang L Li W Chen B Liu Y. cGAS-STING, an Important Pathway in Cancer Immunotherapy. J Hematol Oncol (2020) 13:81. doi: 10.1186/s13045-020-00916-z 32571374 PMC7310007

[27] He C Qiao H Jiang H Sun X. The Inhibitory Role of B7-H4 in Antitumor Immunity: Association With Cancer Progression and Survival. Clin Dev Immunol (2011) 2011:695834. doi: 10.1155/2011/695834 22013483 PMC3195678

[28] Sica GL Choi IH Zhu G Tamada K Wang SD Tamura H. B7-H4, A Molecule of the B7 Family, Negatively Regulates T Cell Immunity. Immunity (2003) 18:849–61. doi: 10.1016/s1074-7613(03)00152-3 12818165

[29] Hao D Li J Wang J Meng Y Zhao Z Zhang C. Non-Classical Estrogen Signaling in Ovarian Cancer Improves Chemo-Sensitivity and Patients Outcome. Theranostics (2019) 9:3952–65. doi: 10.7150/thno.30814 PMC6587348 31281524

[30] Ranganathan P Nadig N Nambiar S. Non-Canonical Estrogen Signaling in Endocrine Resistance. Front Endocrinol (Lausanne) (2019) 10:708. doi: 10.3389/fendo.2019.00708 31749762 PMC6843063

[31] Flynn RL Zou L. ATR: A Master Conductor of Cellular Responses to DNA Replication Stress. Trends Biochem Sci (2011) 36:133–40. doi: 10.1016/j.tibs.2010.09.005 PMC3024454 20947357

[32] Meyuhas O. Ribosomal Protein S6 Phosphorylation: Four Decades of Research. Int Rev Cell Mol Biol (2015) 320:41–73. doi: 10.1016/bs.ircmb.2015.07.006 26614871

[33] Kramer M Dees C Huang J Schlottmann I Palumbo-Zerr K Zerr P. Inhibition of H3K27 Histone Trimethylation Activates Fibroblasts and Induces Fibrosis. Ann Rheum Dis (2013) 72:614–20. doi: 10.1136/annrheumdis-2012-201615 22915621

[34] Kuzet SE Gaggioli C. Fibroblast Activation in Cancer: When Seed Fertilizes Soil. Cell Tissue Res (2016) 365:607–19. doi: 10.1007/s00441-016-2467-x 27474009

[35] Chan MM Tahan SR. Low-Affinity Nerve Growth Factor Receptor (P75 NGFR) as a Marker of Perineural Invasion in Malignant Melanomas. J Cutan Pathol (2010) 37:336–43. doi: 10.1111/j.1600-0560.2009.01349.x 19615036

[36] Keren L Bosse M Marquez D Angoshtari R Jain S Varma S. A Structured Tumor-Immune Microenvironment in Triple Negative Breast Cancer Revealed by Multiplexed Ion Beam Imaging. Cell (2018) 174:1373–87.e1319. doi: 10.1016/j.cell.2018.08.039 30193111 PMC6132072

[37] Thibult ML Mamessier E Gertner-Dardenne J Pastor S Just-Landi S Xerri L. PD-1 Is a Novel Regulator of Human B-Cell Activation. Int Immunol (2013) 25:129–37. doi: 10.1093/intimm/dxs098 23087177

[38] Ahn E Araki K Hashimoto M Li W Riley JL Cheung J. Role of PD-1 During Effector CD8 T Cell Differentiation. Proc Natl Acad Sci USA (2018) 115:4749–54. doi: 10.1073/pnas.1718217115 PMC5939075 29654146

[39] Serres E Debarbieux F Stanchi F Maggiorella L Grall D Turchi L. Fibronectin Expression in Glioblastomas Promotes Cell Cohesion, Collective Invasion of Basement Membrane *In Vitro* and Orthotopic Tumor Growth in Mice. Oncogene (2014) 33:3451–62. doi: 10.1038/onc.2013.305 23912459

[40] Jeevan DS Cooper JB Braun A Murali R Jhanwar-Uniyal M. Molecular Pathways Mediating Metastases to the Brain *via* Epithelial-To-Mesenchymal Transition: Genes, Proteins, and Functional Analysis. Anticancer Res (2016) 36:523–32. 26851006

[41] Fares J Ulasov I Timashev P Lesniak MS. Emerging Principles of Brain Immunology and Immune Checkpoint Blockade in Brain Metastases. Brain (2021) 144:1046–66. doi: 10.1093/brain/awab012 PMC8105040 33893488

